# Understanding the self-management experiences and support needs during pregnancy among women with pre-existing diabetes: a qualitative descriptive study

**DOI:** 10.1186/s12884-023-05542-4

**Published:** 2023-05-02

**Authors:** Katelyn Sushko, Patricia Strachan, Michelle Butt, Kara A. Nerenberg, Diana Sherifali

**Affiliations:** 1 Faculty of Health Sciences, School of Nursing, McMaster University, 1280 Main Street West, Hamilton, ON L8S 4K1 Canada; 2 Department of Medicine, University of Calgary, Calgary, AB Canada; 3 Diabetes Care and Research Program, Hamilton Health Sciences, Hamilton, ON Canada

**Keywords:** Type 1 diabetes, Type 2 diabetes, Pregnancy in diabetics, Self-management, Qualitative research

## Abstract

**Background:**

With the increasing prevalence of pre-existing type 1 and type 2 diabetes in pregnancy and their associated perinatal risks, there is a need to focus on interventions to achieve optimal maternal glycemia to improve pregnancy outcomes. One strategy focuses on improving diabetes self-management education and support for expectant mothers with diabetes. This study’s objective is to describe the experience of managing diabetes during pregnancy and identify the diabetes self-management education and support needs during pregnancy among women with type 1 and type 2 diabetes.

**Methods:**

Using a qualitative descriptive study design, we conducted semi-structured interviews with 12 women with pre-existing type 1 or 2 diabetes in pregnancy (type 1 diabetes, n = 6; type 2 diabetes, n = 6). We employed conventional content analyses to derive codes and categories directly from the data.

**Results:**

Four themes were identified that related to the experiences of managing pre-existing diabetes in pregnancy; four others were related to the self-management support needs in this population. Women with diabetes described their experiences of pregnancy as terrifying, isolating, mentally exhausting and accompanied by a loss of control. Self-management support needs reported included healthcare that is individualized, inclusive of mental health support and support from peers and the healthcare team.

**Conclusions:**

Women with diabetes in pregnancy experience feelings of fear, isolation and a loss of control, which may be improved through personalized management protocols that avoid “painting everybody with the same brush” as well as peer support. Further examination of these simple interventions may yield important impacts on women’s experience and sense of connection.

**Supplementary Information:**

The online version contains supplementary material available at 10.1186/s12884-023-05542-4.

## Background

### Introduction

The global prevalence of diabetes is increasing rapidly. Recent estimates place the current number of people affected by diabetes at 424.9 million, representing 8.8% of the world’s population [1]. In tandem, there has been a rise in the occurrence of type 1 and type 2 diabetes in pregnancy [2–7], which presents serious risks to maternal-child health, including congenital anomalies, stillbirths, and maternal death [2]. As evidence suggests that high glucose levels play an important role in these complications [8, 9], recent attention has focused on improving diabetes self-management education and support for expectant mothers with diabetes [10].

Diabetes self-management education and support is a cornerstone of diabetes management that empowers patients’ active participation in their diabetes management [11]. Self-management is a continuous process, accomplished through various means with tailoring to the patient’s knowledge, skills and abilities [11, 12]. The goal is to improve cardiometabolic and quality of life outcomes [11, 13]. Through self-management education, patients take on an active role in their care with support from their healthcare team [11]. For example, patients may trend their blood glucose measurements and self-adjust their insulin administration to better reach their target range. Offering diabetes self-management education and support is particularly important during times of life changes [14]. Critical changes include when a child transitions to adult diabetes services [14] or when planning and becoming pregnant, among others. Diabetes educators play a major role in providing diabetes self-management education. Clinical practice guidelines suggest that self-management education should be supplemented by self-management support tools, which enhance and reinforce education, and may include text messages, email reminders, automatic phone reminders, peer support and mobile health interventions [11–14]. Such strategies are utilized to improve patient self-efficacy, confidence, and ability to effectively self-manage diabetes [11].

Pregnant women with type 1 and type 2 diabetes experience regular strain and burden associated with daily self-management, involving frequent self-monitoring of blood glucose, accurate titration of insulin doses to blood glucose measures and carbohydrate intake, and close health monitoring [11]. Although sharing similar self-management tasks during pregnancy, women with type 1 and type 2 diabetes may have different experiences of self-management. For instance, women with type 2 diabetes could have been recently diagnosed, may switch to insulin injections during pregnancy from usual oral medications, and are less likely to have had specialized preconception care and counselling [10]. Although arguably more practiced in self-management, women with type 1 diabetes may also face pregnancy-specific challenges, albeit different from those experienced by women with type 2 diabetes. For example, reduced counterregulatory hormones during pregnancy contributes to a high occurrence of hypoglycemia. This is particularly true during the first trimester when severe hypoglycemia may occur in up to 71% of women [10]. Furthermore, increased insulin requirements often occur during pregnancy due to the “anti-insulin” effects of placental hormones later in the second and third trimesters [10].

### Study objective

The purpose of this study was to describe participants’ experiences of managing diabetes during pregnancy and identify the diabetes self-management education and support needs during pregnancy among women with type 1 and type 2 diabetes.

## Methods

### Study design

We used qualitative description as our underpinning methodological qualitative approach. With this qualitative approach, semi-structured interviews and open-ended questions are used to explore the experiences of a purposeful sample of participants [15]. The collected data is analyzed with the intent of allowing the study results to stay as close to the participant’s language as possible. The end result is a rich participant-centered narration regarding their experience with the phenomenon of interest [15]. This study received ethics approval from the local Research Ethics Board (Hamilton Integrated Research Ethics Board Project ID #13,847). Study reporting followed the Consolidated Criteria for Reporting Qualitative Studies framework [16].

### Recruitment and participants

We used purposeful sampling [17] to recruit women 18 years and older with type 1 and type 2 diabetes in pregnancy living in Southern Ontario. We also used snowball sampling to aid in the recruitment of eligible participants. As this study was conducted during the COVID-19 global pandemic, we recruited participants who were currently pregnant or who were recently pregnant (within 12 months prior to recruitment) virtually through advertisements on local and regional pregnancy and diabetes online communities (e.g., Facebook). A total of 12 women with pre-existing diabetes in pregnancy were recruited between March and July 2022 (type 1 diabetes, *n* = 6; type 2 diabetes, *n* = 6). Participants were recruited until thematic saturation was achieved.

### Data collection

The interviews were conducted face-to-face via secure video conferencing software (Zoom) and ranged between 30 to 60 min. Interviews were directed by an interview guide that was informed by a review of the literature and consultation experts in the field of pre-existing diabetes in pregnancy (see the Additional file 1: Appendix). All interviews were audio-recorded and conducted by the first author (KS). Informed consent was acquired and participant demographic information was collected before the interview commenced.

### Data analysis

The audio recordings were transcribed verbatim into Microsoft Word for analysis by the first author (KS). We employed conventional content analyses, as outlined by Hsieh and Shannon, allowing codes and categories to be derived directly from the data rather than from preconceived ideas informed by existing literature or theories [18]. Analysis began following the completion of the first interview with repeated reading of the interview transcripts to facilitate our immersion in the data and to gather an impression of the data as a whole [18]. The first author then derived codes following a word-by- word re-reading of the text and the highlighting of exact words used by participants. Subsequent reviewing of the text and codes first allowed related codes to be grouped into themes, and then into two overarching categories related to the study objectives. The first author (KS) completed an initial analysis. The senior author (DS) then reviewed samples of the transcribed records and conferred with the first author regarding the development of codes, themes and categories throughout the remaining duration of the analysis. Thematic saturation was achieved after ten interviews. The emergence of no new themes was confirmed following the completion of the last two interviews. In addition, the first author, an experienced healthcare provider in the field of maternal-child health, and the senior author, an experienced qualitative researcher and expert on the topic of diabetes self-management education and support, concurred that the findings were credible and confirmable.

### Rigour and trustworthiness

We took several steps to ensure trustworthiness in this study. The interviewer spent a prolonged time in the interview stage and completed multiple interviews to ensure credibility. Triangulation was also used to verify the data sources of interview notes against the actual interview transcripts. The concurrent data collection and iterative analysis also served as a verification method for trustworthiness in this study. Transferability was facilitated through a clear description of the study participants, as well as the methods used to sample the participants. The collection of characteristics of included participants (demographic and clinical characteristics) also supported the transferability of findings to other populations. The study processes were also documented through field notes, interview guides, recorded interviews and data analysis and interpretation notes. Finally, the first author (KS) engaged in self-reflection by writing a reflexivity statement in order to make clear their positionality as a qualitative researcher [19].

## Results

Twelve women (see Table 1 for demographics) participated in individual semi-structured interviews to describe their experience of managing diabetes and their needs regarding self-management education and support during pregnancy. From these interviews, we identified a total of eight qualitative themes. As per the study’s purpose, we organized the themes into two overarching categories – (1) themes describing patient experiences of managing diabetes in pregnancy; and (2) themes identifying suggested needs for diabetes self-management education and support during pregnancy.

**Table 1:** Participant characteristics

Characteristic	Mean (SD)	n [%]
Age (years)	36 (5)	
Ethnicity		
European		7 [58]
Asian		2 [17]
Middle Eastern		1 [8]
African or Caribbean		2 [17]
Indigenous		
Education		
Grade school		
High school		3 [25]
College/trade		
University		9 [75]
Annual household income		
< $20,000		1 [8]
$20,000—$40,000		
$41,000—$60,000		
$61,000—$80,000		
$81,000—$100,000		2 [17]
> $100,000		9 [75]
Diabetes type		
Type 1		6 [50]
Type 2		6 [50]
Diabetes duration (years)		
Type 1	19 (11)	
Type 2	2.5 (1)	
Diabetes treatment method		
Oral medications		
Oral medications and insulin injections		6 [50]
Insulin injections		1 [8]
Insulin pump		5 [42]
Diabetes complications		
Neuropathy		1 [8]
Retinopathy		1 [8]
Singleton pregnancy		12 [100]
Use of Assisted Reproductive Technology		3 [25]

*SD* standard deviation

### Experience of diabetes self-management in pregnancy

#### Theme 1: Terrifying

Women with type 1 diabetes had experienced intensive counselling regarding the need for optimal glycemia before and during pregnancy to avoid having a baby that was ‘gigantic’ or having a baby that was 'at risk of dying.’ Those with type 2 diabetes were often grappling with a newer diabetes diagnosis, as compared to their counterparts with type 1, and feared how this condition, which they were relatively unfamiliar with, would impact their baby's health. Thus, one of the most common things that participants described was fearing the medical complications that could happen to their baby due to diabetes during pregnancy.

“*It [pregnancy] was probably one of the most challenging times of my life in managing diabetes because of that background fear that something bad was going to happen*.”

[Participant 2, type 1 diabetes]

Another participant discussed her feelings as,

“*You feel guilty when your blood sugar is high… you're like*,

‘*What important body part is being formed right now? And am I ruining it*?’”

[Participant 6, type 2 diabetes]

Understanding from the healthcare team that running ‘very, very tight’ was the best way to reduce complications, any small period of hyperglycemia was enough to induce terror, even for those women whose glycemia was near ‘perfect.’ There was an aspect of feeling ‘guilty’ and blaming themselves for the occurrence of these feared complications. Women rationalized their self-blame by suggesting that their baby ‘didn’t ask for this [diabetes].’ Thus, they wanted to do everything in their power to not just be doing ‘great’ with diabetes management but to be actually ‘eliminating the risk’ of complications. One participant described the cause of her fear:

*“I think that some healthcare practitioners who are not well-versed in talking to people with diabetes about complications and babies inadvertently scare them... I think that a lot of them should probably receive additional training in how to talk to women without scaring them and making them worry that everything will be their fault… Most of us are really aware… [we] reach out and learn as much as [we] can before [we] become pregnant.”*

[Participant 1, type 1 diabetes]

Finally, a participant discussed how reading medical journals further emphasized her fears:

*“It’s constant worry and anxiety that you know I'm messing something up for this…kiddo… I go on and find you know what the academic journal articles say about risks and like it's just awful.”*

[Participant 5, type 2 diabetes]

#### Theme 2: Out of control

Due to their feelings of fear and guilt regarding the effect of diabetes on their baby, participants with both type 1 and type 2 diabetes described the need to control their diabetes management as much as possible, including bargaining with physicians and healthcare providers to remain on the devices (i.e., insulin pumps) that give women autonomy and control of their diabetes management.

*“I had, you know built up the courage 'cause you're like, ‘I'm gonna tell this doctor how I want things done’ and so I was like ‘I want to be in charge. I don't want to take off my pump like under no circumstances.’ I was like ‘I will teach my husband to use it before I am taking it off.’”*

[Participant 7, type 1 diabetes]

Another participant articulated the following:

“*Very early on I was asking like ‘I want to control my diabetes at the end.’’… and they were like ‘That's not what we do here. The protocol here is you will be put on an insulin IV*’…

*Just basically flat out told me ‘No that’s not what we do here*.’

…*If I pushed back it was always like ‘We have to do this or your baby's at risk of dying. You're at risk of dying.’ So then how do you as a mother like advocate and ask questions when the answer is like ‘If you don't do what we're telling you to do, your baby will probably die and it will be your fault’”*?

[Participant 10, type 1 diabetes]

Outside of pregnancy, many of the women were used to having total control of their blood glucose. They revealed the great lengths that they went to determine accurate carbohydrate counts for their meals when they could not eat at home due to career demands, for example. Some women pored over nutrition information from restaurant websites. Others took the ‘exact same’ meals to work every day with the carbohydrate count permanently marked on the containers. These actions resulted in their blood glucose being in the ‘non-diabetic or very minimally-diabetic range’ during pregnancy. Having experienced being in full control of their glycemic management before and during pregnancy, participants conveyed their desire to be in control of their diabetes management during labour and delivery. Unfortunately, they frequently received pushback from their healthcare team. With glucose management out of their control, women worried about the complications that could happen to themselves and their babies if hyperglycemia occurred during labour. Thus, they made great efforts to remain in control, resorting to giving themselves their insulin from home, without the knowledge of their healthcare team. A participant noted the following:

*“You had to surrender a lot of control, because… they put you on an insulin drip [during labour]… but afterwards they… stopped the insulin drip too early and so I had ridiculously high blood sugars and they wouldn't sign over control back to me… So needless to say, I had my own pump that I ran myself and gave myself a bolus of insulin… it would have been a potential DKA admission.”*

[Participant 2, type 1 diabetes]

Finally, one mother expressed her concern about control and autonomy related to her diabetes management as:

*“I told my Endo[crinologist], you know, ‘I know that normal protocol is to go on IV Insulin. Never in a million years will I let another person touch my blood sugars. Please, please write the little note that I am going to be managing all of that myself in the hospital. No one is touching my blood sugars.’”*

[Participant 1, type 1 diabetes]

Many of the described self-management behaviours, such as determining accurate carbohydrate counts for meals, apply to women with type 1 diabetes. However, for women with type 2 diabetes that are on insulin during pregnancy, they also expressed similarly going to great lengths to ensure optimal glycemia. One participant described:

*“At work sometimes I would check my blood sugar in a meeting. Because I was very like concerned about like not getting like the data and I was like ‘Well too bad.’ Like I'm gonna, I just put my hand under the table and I would just like do it and whatever get the reading and and then yeah at lunchtime again I would take my insulin and then I would make sure that I eat my lunch 15 minutes later.”*

[Participant 11, type 2 diabetes]

#### Theme 3: Isolating

Perhaps as a result of receiving such close medical monitoring during pregnancy compared to their peers, as well as the immense effort that they had to put into maintaining optimal glycemia, participants relayed that the experience of having diabetes in pregnancy made them feel isolated and different from other expectant mothers without diabetes. For example, women revealed having to do extensive preparation when doing simple outings like going for a walk or going out with friends who didn’t have diabetes.

“It's like I can't, I feel like I can't be a normal person because I'm constantly having to check my blood sugar, constantly having to remember to take my insulin or if I’m going out like I have to make sure ‘OK do you have your insulin? Do you have your meter just in case your sensor goes wrong?’… I just I wish I was a normal person, but I'm not.”

[Participant 4, type 2 diabetes]

“*I had never done this before and I didn't know anyone else that was pregnant that had diabetes*…”

[Participant 2, type 1 diabetes]

Thus, participants reported attempting to seek out others with diabetes in their area with whom to connect.

“*It would have been nice to have, like a pregnant diabetic friend. I've never had one of those*…

*There was this woman in my old neighborhood… and she obviously had diabetes. The way I knew was because before she wore a CGM, I could hear the test strips bouncing in her bag. And then she started wearing a CGM and she was a runner. So I called her the diabetic runner. I used to joke to my husband that I was going to follow her and find her*.”

[Participant 7, type 1 diabetes]

Unfortunately, they did not have much success in connecting with other expectant mothers with diabetes. In addition, women with diabetes in pregnancy felt isolated when with others with diabetes who were not pregnant. Some participants described attending a diabetes education class or a support group and feeling like they didn’t fit in with the other attendees, who they perceived as less advanced in diabetes management. Thus, they experienced a paradox of being isolated with those without diabetes and when with others with diabetes. Women described this as:

“They make you like do these you know pump groups to learn about pumps and carb counting and all this and I've had to do them somewhat recently and it's like ‘OK, I'm seeing people that don’t have the same experience as me. You know, newly diagnosed or whatever and its mind-numbing.’”

[Participant 5, type 2 diabetes]

“I went to a diabetes support group once and I hated it, like hated it. There was one really cool old man who had had diabetes for like 60 years. And then everybody else, it was just like these stories about like one woman who lost her driver’s license because she was low and hit someone. And like someone was losing their vision and then like this, is not, these are not my people.”

[Participant 7, type 1 diabetes]

#### Theme 4: Mentally exhausting

Finally, participants reported that managing diabetes during pregnancy took a toll on their mental health. The tasks that they had to complete related to diabetes self-management were numerous. These included, but were not limited to, planning meals with their carbohydrate count in mind when going to work and when attending social events; measuring their blood glucose at frequent intervals throughout the day, even ‘under the table’ at work during meetings; and taking time off work at least once a week towards the end of pregnancy for medical appointments, sometimes driving long distances to the tertiary center where their medical care occurred if they lived further away.

“I think from a stress standpoint, like it was very tough because I was just learning how to be pregnant and then there was like this extra pressure because it was like a high-risk pregnancy and then on top of that I'm now trying to learn how to manage like diabetes and the monitoring. Like making sure that I was checking my blood sugars like two hours after I ate, making sure I was eating.”

[Participant 11, type 2 diabetes]

“I think like my mental health is definitely a big challenge in terms of my diabetes. Because, like I have a history of like anxiety and depression and being diabetic on its own is stressful enough to me… and then being diabetic and pregnant, taking an even bigger toll on my mental health… Like sometimes I'll I get overwhelmed and it's like I don't care anymore, …I'm just like, ‘I know I'm defeated. I don't want to do this anymore.’ … my mental health and my diabetes definitely, they definitely fight a lot in my head.”

[Participant 4, type 2 diabetes]

One woman even revealed needing to coordinate having someone drive her since the hospital was a long distance away and she was worried about having a hypoglycemic episode and needing to stop at the side of the highway, something she had experienced before. All of this took a significant toll on women’s mental health. A participant’s quotes demonstrating this theme can be found below:

*“Everyone’s experience will be very different. But for someone like myself who has to do it every day, anyway, I mean the burnout is real … sometimes I just look at my husband like ‘Diabetes! I just want a week of vacation.’”*

[Participant 1, type 1 diabetes]

### Patient-identified diabetes self-management support needs in pregnancy

#### Theme 1: Care needs to be Individualized

Since women with type 1 and 2 diabetes have a high-risk pregnancy, they require close medical monitoring at a tertiary center. This means that they undergo frequent testing, such as ultrasounds, sometimes without individualizing care needs and issues.

*“You need to like talk to the person beforehand rather than just going through your like predefined list of all the thing. I think like all principles of adult education, like, asking first, ‘Is this something you would like me to talk about? Are you aware of this? And I think that needs to be included more in initial conversations by healthcare providers.”*

[Participant 1, type 1 diabetes].

Although some of the testing may be required to ensure fetal well-being, such as measuring fetal growth, participant’s described other testing as being done ‘to decide whether or not I want to continue with the pregnancy [should the baby have Down Syndrome].’ After stating she didn’t want a certain test as she would be continuing with the pregnancy regardless, she was told ‘Well, that's, you know that's part of it, you're getting that test.’ The participant was frustrated expressing ‘I still had the test, right like so my lack of ability to like choose and even be informed if I hadn't asked…’ Experiences like these prompted participants to desire their clinical care to feel individualized and tailored to their unique needs, not having things done just because it was protocol. Participant quotes demonstrating this theme can be found below:

*“What remains my biggest thing with like diabetes care in pregnancy is that they sort of like treat everybody as if they're all the same level of risk, when in fact the risk is very different based on what's actually happening with each individual person. And so yeah, I just wish there was a, uh, a better way for people to feel like they're being treated like their own person. And like all of their circumstances, are being taken into account and not just ‘You have type one diabetes so your baby is going to be giant. They're going to have a low blood sugar and you have terrible control…* “*‘Well, we just paint everybody with the same brush because we don't want anybody slipping through the cracks.’ And for me I was like, ‘Well, what if you just actually paid attention to everybody so that they didn't slip through the cracks?’”*

[Participant 10, type 1 diabetes]

#### Theme 2: Peer support

As noted in one of the previously mentioned themes, the experience of diabetes in pregnancy was isolating. Participants reported not fitting in with pregnant women or mothers without diabetes as well as not fitting in with non-pregnant individuals with diabetes.

*“My best friend is great. She's wonderful …I can go to her and I can be like, ‘Oh my, my blood sugars are all over the place and everything’ but all she's gonna say is ‘Oh, you know, like you got this, you can do it’ or you know,’ Talk to your doctor.’ Like she's just gonna be that like little bit of support whereas like I need someone that's like gone through it… I love my best friend but I need a diabetic best friend to go through this with me… like I'd rather have someone be like ‘Bro, I know it sucks, but we got this like we can do this.’ Not just being like ‘You got this’ saying like ‘We have this’ so I know that like there's somebody else in my corner, that's actually going through this and understands everything…”*

[Participant 4, type 2 diabetes]

Thus, stemming from the feelings of isolation during pregnancy with diabetes, women expressed the need for social support from other women with diabetes who they could ‘share experiences with’ and ‘learn with.’

*“It would have been nice to have that in-person connection to someone else like me… to connect to somebody else who would have been, like you know someone who's not just on Facebook. That would have been nice… If they offered me that while I was pregnant that would have been good.”*

[Participant 1, type 1 diabetes]

One participant described reading a book about diabetes in pregnancy with each chapter correlating to a different week in pregnancy. Although she found this helpful, she desired a ‘diabetic friend’ to ‘bounce ideas off of’ when managing diabetes and pregnancy was challenging. Another woman described reaching out to online communities for troubleshooting when her insulin requirements became too high for her pump sensor sites. Having in-person support from someone who understood what she was going through would have been helpful rather than the ‘trial and error’ way that she solved it. A participant described the following:

*“Here I am pregnant and there's gotta be people like me. And I've asked multiple times about like if there's anyone on your caseload that is asking the same like please pass my name along… Like I'm happy to share that experience, or at least be a sounding board. And it's been ‘Well, when you're at the clinic, you know when you come in on Thursdays 'cause that's when the diabetic moms are in, don't hesitate to talk to someone in the waiting room’ and I was like ‘No, I don’t know that I’m going to do that cause not everybody necessarily wants to do that.’ But yeah. It seems like a real missed opportunity…”*

[Participant 5, type 2 diabetes]

#### Theme 3: Mental health support

Women described that the mental health burden of daily self-management was high during pregnancy. For women with type 1 diabetes, they were so experienced in their diabetes management that they knew ‘exactly what a skittle would do’ to their blood glucose. However, during pregnancy, they could eat one thing and have a certain response in their blood glucose and the next day eat the same thing and the blood glucose response would be completely different. For women with type 2 diabetes, particularly those who were relatively newly diagnosed, it was difficult to navigate the new diagnosis along with the pregnancy and the addition of insulin to their diabetes management.

“*Sometimes it's not that I need diabetic help, like medical help*. *It's like I just need some mental clearance.”*

[Participant 4, type 2 diabetes]

“The piece that wasn't a part of the high-risk clinic was the mental health piece and… I don't think that people look at it so seriously. And I don't think anybody talked to me about like the fact that like I was feeling stressed out… So I think that that was something that was missing.”

[Participant 11, type 2 diabetes]

Their pregnancies also occurred during the COVID-19 pandemic. Thus, they had to attend their medical appointments alone, without their partner, due to hospital policies. These factors, among others, culminated in women expressing the need for mental health support.

“It was really hard and especially… 'cause my husband was back home and I'm alone here… I didn't eat so I lost lots of weight because, you know, stressed out. Every day I'm crying, I'm emotionally – it’s not easy.”

[Participant 3, type 2 diabetes]

#### Theme 4: Support from healthcare team

Finally, participants expressed the need for support for diabetes self-management from the healthcare team. Support from the healthcare team was desired for a variety of instances. We developed two subthemes to represent two of the common instances: (1) support in self-management of diabetes during the labour and delivery process; and (2) supportive health professional communication. One of the experiences expressed by several participants with type 1 diabetes, was the desire to control their insulin management during labour and delivery (described in a previous theme). However, women expressed that they were not supported by their healthcare team in this area. More than one participant explained that they kept themselves in ‘non-diabetic’ glucose ranges before and during pregnancy, without experiencing hypoglycemia. Nevertheless, during labour and delivery, they experienced hyperglycemia when insulin management was done by the healthcare team. One woman described her experience as follows:

*“I could have kept myself between 5 and 6 and you [healthcare team] took over and you did not. I was like over 10 for much of it and at one point like my husband was like calling the OB and like literally yelling at him on the phone. ‘Why aren't they listening? Why can't we just give like a bolus to bring her blood sugar down, it's like been over 10 for an hour. Like can we at least give a bolus?’ And it was like, ‘Nope, we're just going to follow the protocol,’ which is not based even on me. It's like they give this same amount for everyone… It makes no sense.*

[Participant 10, type 1 diabetes]

She managed to convince the team to let her control her insulin during her second labour and delivery but was surprised when they would not help her determine appropriate pump settings before delivery and she was left to fend for herself.

*““I remember just asking like ‘All I want from you [endocrinologist] is like a suggestion on where my insulin like starting point should be. Should I be basing it on what I'm taking now? Should I be basing it on what I was taking pre-pregnancy?’ And he just was like ‘Well that’s up to you.’ Like he wouldn’t help me at all… I was so dumbfounded that like he was sort of like ‘Fine, you can be in control, but I'm not going to help you… I reached out to my community, to kind of get a read on what I thought was pretty standard [pump settings for labour and delivery] and just I tried to figure it out on my own…”*

[Participant 10, type 1 diabetes]

Thus, more support from the healthcare team was a general theme that many women expressed. Another participant also expressed the need for support, with emphasis on how healthcare providers communicate with women with diabetes in pregnancy:

*“After I had the baby… the breastfeeding topic comes up, and I elected not to because I was tired of low blood sugars… Nine months of extreme lows that I've had already, you know between pregnancies, and I got pregnant back-to-back all three times. So, I was like, ‘No more, I don’t even want to deal with that. I just want to let the hormones go back to normal.’ Babies can live on formula I did, I'm fine. Um, but the way they talked to you after was just really, I can’t even remember what he said, I remember thinking ‘This guy has never learned how to talk to women about this topic.’ What he should have done is come in and go ‘Would you like me to talk to you about it?’ Cause chances are, most of us have already decided what we're going to do. He didn't even ask my reasons for not breastfeeding. He just went on about how I should be.”*

[Participant 1, type 1 diabetes]

## Discussion

Our study described the participant experiences of managing type 1 and 2 diabetes in pregnancy and identified self-management support needs of this population. In our exploration of this topic, three main areas to improve the experience of these women stood out. These are: (1) early facilitated connection with other moms also experiencing type 1 and type 2 diabetes in pregnancy; (2) clinical management pathways to allow for self-management of diabetes during the labour and delivery process; and (3) health professional communication with women with pre-existing diabetes in pregnancy.

Peer support is means by which people with similar experiences give and receive support related to their shared experiences [20]. It was first pioneered over 40 years ago in the mental health field to increase coping abilities and reduce depression and anxiety [21, 22]. Peer support has also existed as an intervention in the literature on diabetes self-management for several years. National guidelines have included peer support as a component of diabetes self-management support [11]. Self-management support in combination with diabetes self-management education have demonstrated advantages for both optimized glycemia and reduced diabetes distress [11]. A meta-analysis showed that peer support, as a form of diabetes self-management support, among adults with type 2 diabetes may reduce A1C by an average of -0.57% [23]. The existing literature on peer support in diabetes and pregnancy is limited. Friedman et al. conducted a needs assessment for peer support in the postpartum period for women with gestational diabetes and type 2 diabetes [24]. This study found that nearly half of the participants were interested in a Diabetes Buddy Program [24]. A qualitative study involving women with previous gestational diabetes found peer support instrumental in healthy behaviour changes [25]. Two studies, one by Berg et al. and one by Linden et al., described the development of a web-based support program that contained an element of online peer support and its subsequent evaluation in a randomized controlled trial [26, 27]. The randomized controlled trial found that active participation in the web-based program was low and resulted in no differences in well-being or diabetes self-management. Participants in our study emphasized their desire for peer support in person. Thus, perhaps participation would have been more active had the peer support not been virtual. Further research is needed to advance the evidence supporting this type of intervention.

Women described intense resistance from the healthcare team regarding self-management of diabetes, with their insulin pump, particularly during labour. However, outside of pregnancy, it is common practice for adults with diabetes to self-manage insulin administration during medical procedures, such as short surgeries [28]. Guidelines and protocols for diabetes self-management using insulin pumps in the perioperative period were established by the Joint British Diabetes Society in 2011 [29]. Protocols also exist in parts of the United States, Australia and Canada [30–32]. Research on insulin pump therapy during labour has shown that it is safe and may contribute to improved optimized glycemia [33–35]. Women in our study described their desire to control insulin administration during labour and adverse outcomes due to poor management by their healthcare team. Future research should focus on advancing the evidence on this topic to allow women to remain in control of their diabetes management during labour and delivery.

Women articulated multiple instances of poor communication from healthcare providers. These ranged from how potential pregnancy complications related to diabetes were discussed to the way advice or lack thereof was delivered regarding insulin pump settings during labour, among others. Communication between healthcare providers and those with diabetes has been established as an important factor that influences diabetes outcomes [36]. Communication styles that are patient-centered are linked to better self-care and quality of life, for example, among adults with diabetes [37]. Unfortunately, evidence suggests that communication from healthcare provider to patient is often not patient-centered [37]. Evidence indicates that best practice for healthcare provider-patient communication includes: (1) fostering the relationship by building rapport and connection; (2) gathering information by attempting to understand the patient needs; (3) providing information in response to patient needs; (4) engaging in collaborative decision making; (5) enabling disease- and treatment related behaviour, such as self-management; and (6) responding to patient expression of emotion related to their condition [38]. Engaging in a communication style that reflect these practices will contribute to improved positive patient outcomes [38].

Of the three areas that we identified to improve the experience of managing diabetes in pregnancy, the literature indicates that at least two of them are also applicable to women diagnosed with gestational diabetes during pregnancy. These include the desire for peer support and the need for patient-centered communication from healthcare providers. As previously mentioned, women with diabetes in pregnancy, including gestational diabetes, have indicated interest in peer support interventions [24]. Furthermore, women with previous gestational diabetes have expressed that peer support influenced healthy behaviour change [25]. To our knowledge, these are the only studies that have examined the use of peer support among women with gestational diabetes. Furthermore, a recent systematic review of reviews identified the use of peer support on gestational diabetes as a research gap [39]. Regarding healthcare provider communication with women with gestational diabetes, research reveals shortcomings reported by women. For example, women have reported communication with healthcare providers to be difficult and confusing, expressing that they desire a non-judgemental and patient-centered approach [40]. Thus, it appears that an exploration of peer support and improving healthcare provider communication is also warranted for women gestational diabetes by investigators who are focused on this population.

### Strengths and limitations

This study has several strengths. First, we sampled women from a diverse geographic region of Southern Ontario. Thus, the resulting participants varied in terms of ethnicity, education and other sociodemographic factors. In addition, we achieved an equal number of women with type 1 and 2 diabetes, further contributing to the diversity and richness of experience. However, our study also has limitations. Although our participants were socioeconomically diverse in some respects (ethnicity), the majority were well educated and had a high household income. Furthermore, the sample size was relatively small. Thus, like any qualitative research, the generalizability of our results to other populations is limited.

## Conclusions

This study found that the experience of managing diabetes during pregnancy was terrifying, isolating and mentally exhausting, leaving the woman with diabetes feeling out of control. Furthermore, women with diabetes during pregnancy expressed the need for individualized care, social and mental health support and support from their healthcare team in all aspects of diabetes management. To adequately support women with diabetes during pregnancy, policy and decision-makers should re-evaluate current clinical management protocols and consider the use of patient self-management of insulin administration, particularly during labour and delivery. Furthermore, facilitating peer support between expectant mothers is desired and has the potential to reduce feelings of isolation and increase feelings of connection and support. These simple interventions may have important impacts on the pregnancy experience of women with type 1 and 2 diabetes.

## Supplementary Information

**Additional file 1.**  Interview guide.

## Data Availability

Study participants were advised that their raw data would remain confidential and not be shared publicly due to the sensitive nature of the interview questions. Upon reasonable request, the data are available from the corresponding author.
