# Supplementary material for: Understanding the self-management experiences and support needs during pregnancy among women with pre-existing diabetes: a qualitative descriptive study
Source: BMC Pregnancy Childbirth. 2023 May 2;23:309. doi: 10.1186/s12884-023-05542-4 (PMC10152436; doi:10.1186/s12884-023-05542-4)
Supplement: Supplementary file 1 — Additional file 1. Interview guide. [file 12884_2023_5542_MOESM1_ESM.docx]

Appendix – Interview Guide

**Opening Question**

Can you tell me about your day-to-day experience of managing diabetes during pregnancy?

**Main Questions**

Did your experience of managing diabetes during pregnancy differ from how you managed your diabetes before pregnancy?

How important was it to manage your diabetes in pregnancy?

Was there any social, employment, or financial factors that challenged how you managed your diabetes in pregnancy?

Were there any other challenges that I have not mentioned?

Can you tell me about your experience of receiving diabetes care and education during pregnancy?

How did the diabetes education and care that you received during pregnancy inform your diabetes management during pregnancy?

Was there anything that you found helpful?

Was there anything that may have been helpful, but was not provided or offered?

In a future pregnancy, what would be the best way that diabetes education could be provided to you? Telephone, in-person, online, phone apps?

**Probing Questions**

Can you tell me about your experience of checking your blood sugar?

Can you tell me about your experience of counting carbohydrates?

Can you tell me about your experience of giving yourself insulin?

How confident do you feel about your ability to do these activities that we just talked about, such as checking your blood sugar, counting carbohydrates, giving yourself insulin?

[If you feel confident], what do you think contributes to this?

[If you do not feel confident], what do you think contributes to this?

[If you do not feel confident], what do you think could help to increase your confidence?

Can you tell me what makes managing diabetes easier for you?

Can you tell me what makes managing diabetes harder for you?

Can you tell me more about […]?

Can you give me an example of that?

Some women noted […], was this your experience?
